# Effectiveness and safety of dolutegravir plus lamivudine in treating HIV in China, including outcomes of patients coinfected with tuberculosis

**DOI:** 10.1097/MD.0000000000038558

**Published:** 2024-07-05

**Authors:** Haohua Hou, Huanhuan Ba, Xinyan Jin, Peipei Luo, Yuan Zhang, Jiajia Li, Juan Jin

**Affiliations:** a Department of Infectious Diseases, Xi’an Eighth’s Hospital, Xi’an, Shaanxi, People’s Republic of China.

**Keywords:** antiretroviral therapy, dolutegravir plus lamivudine, human immunodeficiency virus infection, tuberculosis, viral suppression

## Abstract

Antiretroviral regimens for human immunodeficiency virus (HIV) infection have continuously evolved; however, antiretrovirals can cause severe adverse reactions. Two-drug regimen therapy can decrease lifetime cumulative drug exposure and long-term toxicities associated with multiple antiretrovirals. The preferred 2-drug regimen constitutes dolutegravir (DTG) and lamivudine (3TC). This study determined the rate of virological suppression and incidence of adverse events at week 48 in treatment-naïve people living with HIV initiated on DTG + 3TC. This was a single-center, retrospective, observational study. Treatment-naïve people aged ≥18 years who received at least 1 DTG + 3TC dose between May 2020 and May 2022 were included. Eighty-nine people living with HIV were enrolled. Twenty-five (28.1%) patients with a DTG + 3TC regimen at baseline were analyzed because of comorbidities, and 48% because of concomitant tuberculosis (TB). Viral suppression at 48 weeks was achieved in 91.67% of patients, and TB was well controlled. At week 48, 84 (94.38%) patients had viral loads < 50 copies/mL, and 21 (91.31%) of the 23 participants with a baseline HIV-1-RNA level ≥ 1 × 10^5^ copies/mL achieved virological success. Fifteen (88.23%) of the 17 participants with a baseline CD4 + cell count of <200 cells/µL achieved virological suppression. The median CD4 + cell count change from baseline was 539.5 cells/µL. No significant changes in triglycerides, low-density lipoprotein cholesterol, weight, or creatinine were observed from baseline to 48 weeks. One patient had severe insomnia at 4 weeks. Our findings support the real-world effectiveness and low metabolic impact of DTG + 3TC. Using DTG + 3TC in patients coinfected with TB and HIV has favorable therapeutic outcomes.

**Key points**

1. In this retrospective study, we described 89 PLWH HIV who received dolutegravir (DTG) and lamivudine (3TC).
2. One patient had severe insomnia at 4 weeks.
3. In this study support the real-world effectiveness and low metabolic impact of DTG + 3TC.
4. In this study support using DTG + 3TC in patients coinfected with tuberculosis and HIV has favorable therapeutic outcomes.

## 1. Introduction

With the development of highly active antiretroviral therapy (ART), human immunodeficiency virus (HIV) infection has become a manageable chronic disease.^[1]^ The traditional highly active ART regimen comprises 2 nucleoside reverse transcriptase inhibitors and 1 non-nucleoside reverse transcriptase inhibitor or protease inhibitor. With the emergence of integrase inhibitors, 2 nucleoside reverse transcriptase inhibitors plus 1 integrase inhibitor have become the primary ART for patients with HIV.

Continued HIV drug development and innovation have greatly improved the success of HIV treatment and increased the life expectancy of patients. However, the long-term use of drugs can cause toxic adverse reactions, such as liver and kidney damage, insomnia, dreaminess, weight gain, and dyslipidemia. With the prolongation of illness, reducing drug interactions with increased comorbidities has become an unsolved problem for many patients with HIV. Therefore, researchers continue exploring simplified schemes to reduce drug side effects and interactions, while ensuring the effectiveness of viral suppression. Since second-generation integrase dolutegravir (DTG) emerged, research on simplified DTG-based schemes has significantly progressed. DTG is an effective integrase inhibitor and has been approved for use with lamivudine (3TC) to treat patients infected with HIV-1 and for virological suppression to reduce long-term exposure to ART. In multiple clinical trials and meta-analyses, DTG-based 2-drug regimens (2-DRs) are reportedly non-inferior to 3-drug regimens.^[2–5]^

Tuberculosis (TB) remains the leading cause of death among HIV-infected people worldwide. In 2018, approximately 251,000 people died due to AIDS and TB. According to China’s 2017 statistics, approximately 12,000 new HIV/TB cases and 1,000 deaths were reported.^[2]^ Based on the TB regimen, rifamycin anti-tuberculous drugs interact with various ART drugs, limiting the choice of treatment options for patients coinfected with HIV and TB. Therefore, this study aimed to determine the virological suppression rate and the incidence of adverse events following the use of DTG-based 2-DRs in treatment-naïve patients, while elucidating the TB treatment outcomes of patients coinfected with TB and HIV concomitantly receiving rifabutin (RFB) and DTG + 3TC in Shaanxi, China.

## 2. Materials and methods

### 2.1. Ethical approval and informed consent

This study was approved by the Institutional Review Board of The Xi’an Eighth Hospital (Institutional Review Board authorization number: 20221106) and performed according to the principles of the Declaration of Helsinki. All patients signed an informed consent form to participate in the study, and all data were analyzed anonymously.

### 2.2. Study design

This was a single-center, retrospective, observational study. Patients infected with HIV who underwent treatment with once-daily DTG + 3TC between May 2020 and May 2022 were recruited. After screening, 96 cases were included; the standard DTG and 3TC doses were 50 and 300 mg, respectively. If the patient had a kidney injury, the 3TC dose was adjusted to 150, 100, or 50 mg according to creatinine clearance.

### 2.3. Inclusion and exclusion criteria

The inclusion criteria were HIV infection and age ≥18 years. Antiviral therapy with a DTG + 3TC treatment regimen was started at the research center from May 2020 to May 2022. All patients had baseline HIV-RNA and CD4 + T lymphocyte levels and were followed up for at least 48 weeks. This was a retrospective study; therefore, we did not calculate sample sizes and included all eligible patients during this period.

The exclusion criteria were hepatitis B viral infection, pregnancy, irregular follow-up, lack of laboratory data during follow-up, and death. Laboratory testing time during follow-up was ± 4 weeks.

### 2.4. Data sources

Routine patient follow-ups were conducted at 4, 12, 24, 36, and 48 weeks. Abnormal laboratory results during patient follow-ups increased the frequency of follow-ups. CD4 + count, viral load (VL), blood routine, blood biochemistry, hematology, chest radiography, and other related tests were performed at baseline. Hematology, blood biochemistry, and urine tests were performed at weeks 4 and 36, and CD4+, VL, blood chemistry, hematology, and urine tests were performed at weeks 12, 24, and 48. The patient’s weight was determined at each visit.

All data were sourced from the hospital system. The primary endpoint was the proportion of HIV-RNA < 50 copies/mL at week 48, and the secondary endpoints included changes in body weight, CD4 + cell count, and blood chemistry at 48 weeks compared to baseline values, and the proportion of patients experiencing adverse effects while taking the regimen.

Osteopenia was defined as a T value of ≤−1 during dual-energy X-ray bone mineral density examination. Renal impairment was defined as albuminuria and a decline in estimated glomerular filtration rate to <60 mL/min/1.73 m^2^.

### 2.5. Statistical analyses

Qualitative variables are reported as frequency distributions, whereas quantitative variables are presented as medians with interquartile range (IQR) or mean with SD. The Kolmogorov–Smirnov test was used to determine whether the numerical variables fit the assumption of normality of distribution. Student *t* test was used to compare normally distributed independent variables between the baseline and week 48, whereas the Mann–Whitney *U* test was used for continuous numerical variables that followed a non-normal distribution. Statistical significance was set at *P* < .05. All statistical analyses were performed using SPSS version 21.0 (IBM Corp., Armonk).

## 3. Results

### 3.1. Patient characteristics

**Figure 1.**
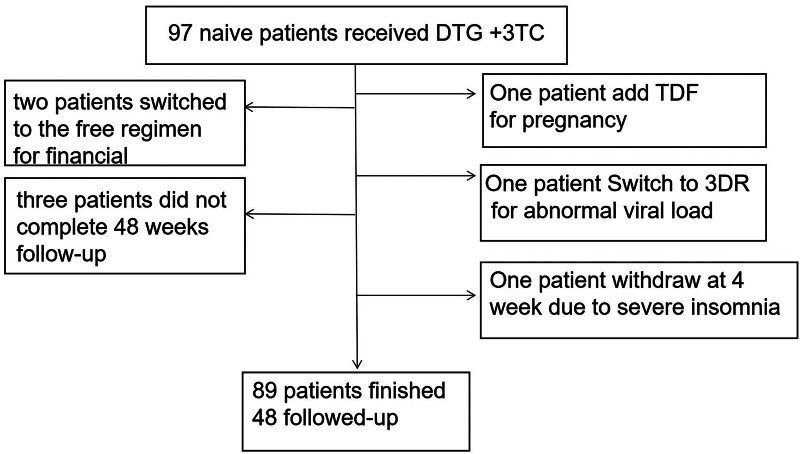
Flow chart for selecting study participants. DTG = dolutegravir, 3TC = lamivudine, TDF = tenofovir disoproxil fumarate, VL = viral load.

A total of 97 patients were treated with DTG + 3TC from May 2020 to May 2022 (Fig. 1). Two patients switched to the free regimen for financial reasons, 1 switched to a 3DR due to pregnancy, 1 switched to a 3DR due to an abnormal VL, and 1 withdrew due to severe insomnia at 4 weeks. Three patients did not complete the 48-week follow-up. Ultimately, 89 patients were included in this study. The baseline patient characteristics are presented in Table 1.

**Table 1:** Baseline characteristics of study participants.

Characteristic	Patients (n = 89)
Sex, n (%)	
Male	85 (95.5%)
Female	4 (4.5%)
Marital status, n (%)	
Single	55 (61.8%)
Married	27 (30.3%)
Divorced	7 (7.9%)
Median age (IQR), yr	46 (3757)
Age ≥ 60 yr, n (%)	7 (7.9%)
Sexual orientation, n (%)	
Homosexual	57 (64.0%)
Heterosexual	20 (22.5%)
Unknown	12 (13.5%)
CD4 + count	
Median (IQR), cells/µL	342 (216,553)
<200 cells/µL, n (%)	17 (19.1%)
≥200 cells/µL, n (%)	72 (80.9%)
HIV VL, copies/mL, n (%)	
<1 × 10^5^	66 (74.2%)
≥1 × 10^5^	23 (25.8%)
ART period, d (%)	
≤14	28 (31.5%)
>14	61 (68.5%)
Comorbidities, n (%)	
Hypertension	4 (4.5%)
Renal impairment	5 (5.6%)
Diabetes	5 (5.6%)
AIDS-related opportunistic infections-pulmonary TB, n (%)	12 (13.5%)
Reason for choosing DTG + 3TC	
Simplification	46 (51.69%)
Renal impairment or high risk	14 (15.73%)
Osteopenia	9 (10.11%)
Reduced drug-drug interaction	20 (22.47%)

3TC = lamivudine, ART = antiretroviral therapy, DTG = dolutegravir, HIV = human immunodeficiency virus, IQR = interquartile range, TB = tuberculosis, VL = viral load.

The study sample was 95.5% male, with a median age of 46 years (IQR, 37–57 years), of whom 7.9% were >60. The median CD4 + number at baseline was 342 cells/µL (IQR, 216–553 cells/µL); 17 patients (19.1%) had a CD4 + count of <200 cells/µL, and the median VL at baseline was 36,150 copies/mL, and 23 patients (25.8%) had a baseline HIV VL > 1 × 10^5^ copies/mL. At baseline, 12 patients (13.48%) had concomitant pulmonary TB. The primary reason for choosing DTG + 3TC was to acquire a simplified treatment regimen (51.69%); 20 patients (22.47%) used DTG + 3TC because of reduced drug–drug interactions. Five patients were tested for drug resistance before treatment; 1 had potential nevirapine and rilpivirine resistance.

### 3.2. Effectiveness of DTG and 3TC

**Figure 2.**
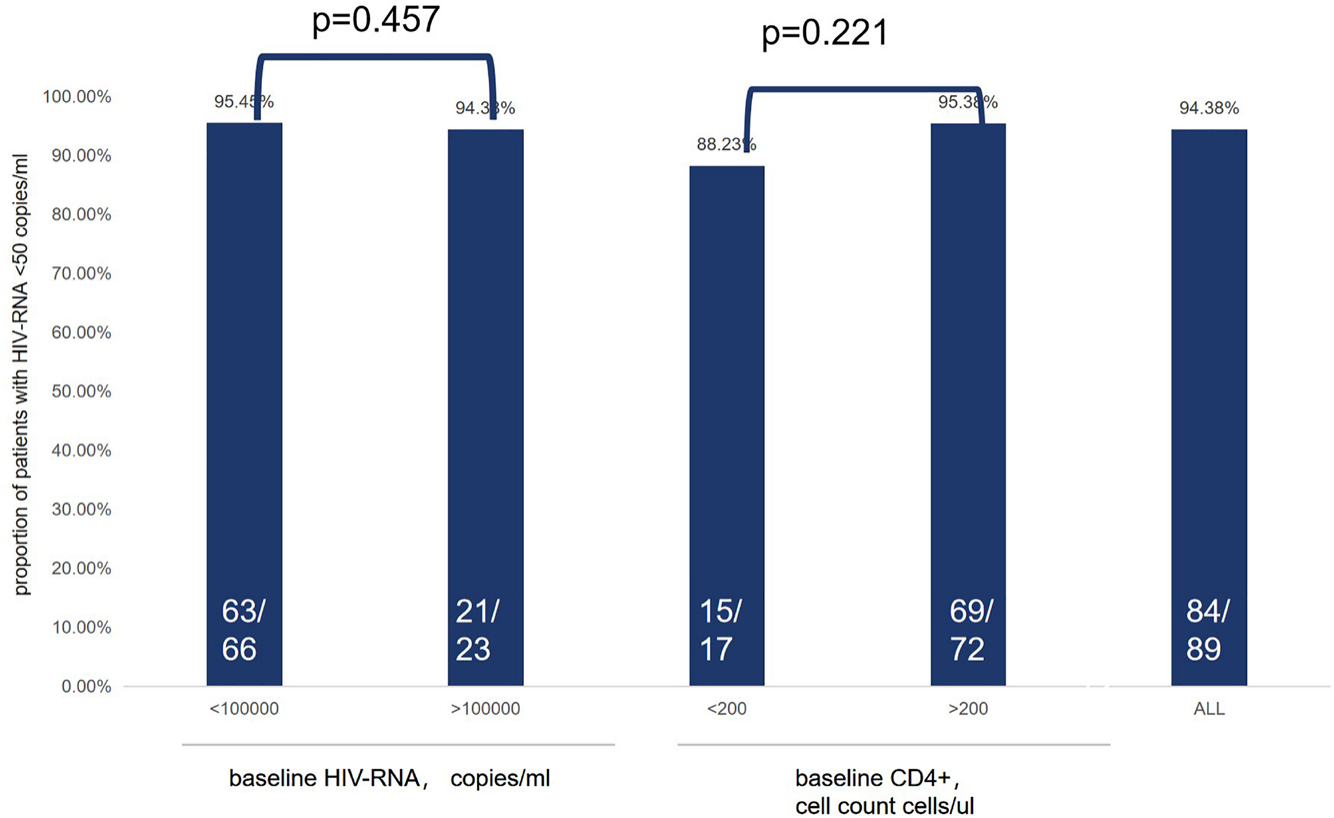
Analysis of patients with an HIV VL of <50 copies/mL at 48 weeks. HIV = human immunodeficiency virus, VL = viral load.

**Figure 3.**
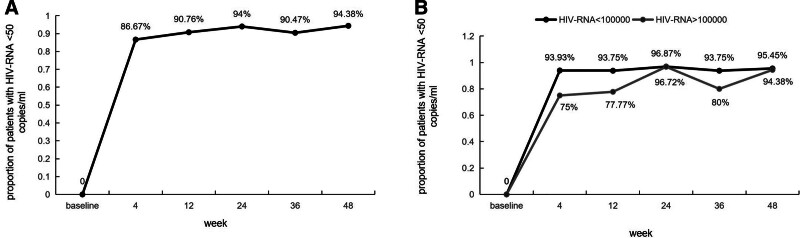
Proportion of (A) patients with plasma HIV-1 RNA levels of 50 copies/mL, with comparable results in (B) the BL HIV-1 RNA levels of <1 × 10^5^ copies/mL and >1 × 10^5^ copies/mL strata. BL = baseline, HIV = human immunodeficiency virus.

At 48 weeks, 94.38% of the participants in this study achieved an HIV-1 VL of <50 copies/mL. The 48-week virological suppression rate of patients with baseline CD4 + cell counts of <200 cells/µL and ≥200 cells/µL were 88.23% and 95.83%, respectively. At baseline, 23 patients (25.8%) had HIV-1 RNA levels ≥ 1 × 10^5^ copies/mL, and 21 patients (91.31%) had HIV-RNA levels < 50 copies/mL after 48 weeks of treatment (Fig. 2). At 24 weeks, the proportion of HIV-RNA levels < 50 copies/mL was 94%. The virological suppression rate at week 24 was 96.72% in the group with HIV-RNA levels > 1 × 10^5^ copies/mL. In the group with HIV-RNA levels ≤ 1 × 10^5^ copies/mL, the virological suppression was 96.87% (Fig. 3). At 48 weeks, 5 patients with HIV-1 VL levels > 50 copies/mL were retested after 4 weeks and had a retest load less than the minimum detection value (VL < 20 copies/mL).

### 3.3. Immune function and safety of DTG and 3TC

**Figure 4.**
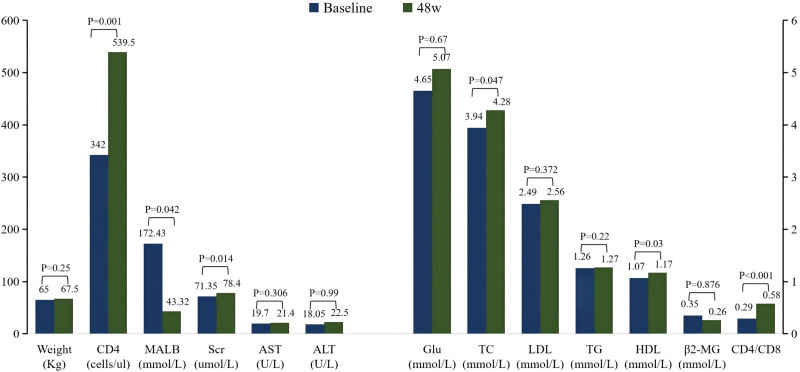
Changes in the immunity and safety indices between baseline and week 48. ALT = alanine aminotransferase, AST = aspartate aminotransferase, GLU = blood glucose, HDL-C = high-density lipoprotein cholesterol, LDL-C = low-density lipoprotein cholesterol, β2-MG = urine β2 microglobulin, MALB = urine microalbumin, Scr = serum creatinine, TC = total cholesterol, TG = triglyceride, UA = uric acid. * expressed as the median (interquartile range) deviation.

The CD4 + cell counts of all patients increased from 342 to 539.5 cells/µL (*P* = .001), and the CD4/CD8 ratio increased by 0.29-fold (*P* < .001) after 48 weeks. We assessed lipid levels, liver function, kidney function, and body weight. The serum creatinine and total cholesterol levels increased, whereas urine microalbumin, uric acid, and high-density lipoprotein cholesterol increased. Urine microalbumin decreased from 72.83 to 58.52 (*P* 00.042). No significant changes in weight, alanine aminotransferase, aspartate aminotransferase, triglycerides, low-density lipoprotein cholesterol (LDL-C), and urine β2 microglobulin were observed (Fig. 4).

### 3.4. Follow-up data of ART-naïve patients with adjusted 3TC doses

Five patients with chronic kidney disease had to adjust their 3TC dose according to the baseline creatinine level and glomerular filtration rate. The dose for 1 patient was adjusted from 150 to 100 mg at 48 weeks (Table 2).

**Table 2:** Follow-up data of ART-naïve patients with adjusted 3TC doses between baseline and week 48.

Number	Age (yr)	3TC dose		Scr (µmol/L)		eGFR (mL/min/1.73 m^2^)		VL (copies/mL)		CD4 + count (cells/µL)	
		Baseline	48 wk	Baseline	48 wk	Baseline	48 wk	Baseline	48 wk	Baseline	48 wk
1	39	150	150	150.8	172.1	49.27	43.7	10800	TND	312	502
2	53	150	150	138.5	141.8	49.72	48.33	41900	28	13	246
3	76	150	150	160.6	175.4	36.89	33.16	357000	TND	320	451
4	66	100	100	321.2	343.04	16.41	15.16	140000	TND	381	378
5	47	150	100	187	282.9	36.08	21.871	3660	TND	264	396

3TC = lamivudine, ART = antiretroviral therapy, eGFR = estimated glomerular filtration rate, Scr = serum creatinine, TND = target not, VL = viral load.

### 3.5. Outcome of DTG + 3TC therapy in patients with TB

During the study period, 12 patients coinfected with TB and HIV received RFB and DTG + 3TC regimens. The mean age was 39.9 years, and 11 patients (91.7%) were male. At 48 weeks, all patients achieved favorable TB treatment outcomes, with a viral suppression rate of 91.67%. The median CD4 + cell count change from baseline was 184 cells/µL ([IQR (69–373)], *P* < .05) (Table 3).

**Table 3:** Characteristics of the study population with TB/HIV coinfection.

Number	Age	TB-site	Anti-TB treatment regimens	TB-outcome	DTG-dose	HIV-RNA levels (copies/mL)		CD4 + counts (cells/µL)	
						Baseline	48 wk	Baseline	48 wk
1	54	Pulmonary	HZE + RFB	Success	50 mg QD	17400	<20	190	359
2	31	Pulmonary	HZE + RFB	Success	50 mg QD	216000	<20	26	181
3	36	Pulmonary	HZE + RFB	Success	50 mg QD	11900	59	277	481
4	37	Pulmonary	HZE + RFB	Success	50 mg QD	47300	<20	278	395
5	43	Pulmonary	HZE + RFB	Success	50 mg QD	31500	<20	212	319
6	58	Pulmonary	HZE + RFB	Success	50 mg QD	305000	<20	48	271
7	25	Pulmonary	HZE + RFB	Success	50 mg QD	1400	TND	379	486
8	48	Pulmonary	HZE + RFB	Success	50 mg QD	99000	<20	36	212
9	49	Pulmonary	HZE + RFB	Success	50 mg QD	36100	<20	10	79
10	35	Pulmonary	HZE + RFB	Success	50 mg QD	51200	<20	245	618
11	28	Pulmonary	HZE + RFB	Success	50 mg QD	46400	<20	243	549
12	40	Pulmonary	HZE + RFB	Success	50 mg QD	21700	TND	213	405

DTG = dolutegravir, HIV = human immunodeficiency virus, HZE = isoniazid + procaine hydrochloride + ethambutol, QD = quaque die, RFB = rifabutin, TB = tuberculosis, TND = target not.

### 3.6. Adverse events

After starting DTG or 3TC, 3 patients complained of side effects. One patient experienced severe insomnia at 4 weeks and changed the ART regimen. Two patients had an increase in alanine aminotransferase up to 2.5 times the normal level for unknown reasons but continued to take the drugs, and their alanine aminotransferase levels eventually returned to normal.

## 4. Discussion

DTG-based 2-DRs are easy to use, have low drug–drug interactions, and are now recommended as first-line HIV ART regimens in several countries.^[4,6–8]^ DTG + 3TC, as a starter regimen for patients infected with HIV, showed good viral suppression with no virological failure. Viral suppression was 94.38% at 48 weeks; 1 patient experienced dizziness and sleep disturbances at 4 weeks, but none discontinued treatment due to adverse events, which is generally consistent with the results of other studies.

The long-term viral suppressive effects of ART drugs are essential for the long-term prognosis of patients infected with HIV-1. The virological results observed in our study were consistent with those of other published clinical trials. In previously published clinical studies, the prevalence of viral suppression at 48 weeks after administering DTG + 3TC to treatment-naïve patients ranged from 85% to 97.2%.^[9–11]^ In our study, the viral suppression rates were 94.38% at 48 weeks, 97.75% at 12 weeks, and 100% at 24 weeks. At 48 weeks, 5 patients with HIV-1 RNA levels > 50 copies/mL but <200 copies/mL were tested again 4 weeks later; all showed VLs < 50 copies/mL following the retest, which we considered a viral blip. Therefore, DTG + 3TC for primary patients can ensure rapid viral suppression, but monitoring the VL regularly is crucial. However, we did not analyze populations with a high disease load, which may be related to the approved indication of DTG + 3TC in China, during the observation period, primarily for patients with HIV with VLs < 500,000 copies/mL.

Regarding immune function recovery, CD4 + T lymphocyte counts < 200 cells/mm^3^ and lower CD4/CD8 levels are strongly associated with the development of opportunistic infections and an increased risk of death in patients with HIV.^[12,13]^ In our study, the CD4 + cell count at 48 weeks increased compared with the baseline, and the CD4/CD8 ratio increased by 0.29-fold. Furthermore, in 17 cases, a significant increase in the CD4 + cell count and CD4/CD8 ratio was observed after DTG + 3TC treatment. A significant improvement in immune function in surface light patients was observed, generally consistent with the results of previous studies.

Drug safety and ease of administration affect patient compliance. Consistent with other findings, at 48 weeks of treatment, patients had elevated creatinine levels without renal function impairment;^[9,14]^ this may be related to DTG inhibiting mailed cation transport protein 2 outside the proximal tubular cell base of the kidney, resulting in a mild, non-progressive serum creatinine elevation.^[15,16]^ This finding is consistent with current clinical findings; therefore, long-term renal follow-up for patients receiving DTG + 3TC is necessary. In this study, 5 patients with 3TC renal insufficiency required dose adjustment. At 48 weeks, no significant change in creatinine and estimated glomerular filtration rate from baseline was observed, the number of CD4 + cells was significantly increased, and DTG + 3TC had a good effect on patients with abnormal renal function and had little effect on the kidneys.

Cardiovascular disease is the most common non-HIV-related disease in patients with HIV, and total cholesterol and elevated LDL-C levels are independent risk factors for cardiovascular diseases.^[17,18]^ In Chinese patients with HIV, the dyslipidemia incidence is 75.6%, primarily manifested by triglyceride and LDL-C increases.^[19]^ Herein, dyslipidemia at 48 weeks was mainly characterized by high-density lipoprotein cholesterol and total cholesterol elevations, and the changes in LDL-C and triglycerides were not significant. However, we observed some differences with the results of a previous study,^[11]^ which may be related to the younger age of the patients included in our study.

Early ART initiation combined with anti-TB therapy can reduce mortality in patients with HIV/TB. Currently, ART for patients with HIV/TB is primarily based on a 3-drug regimen. This study provides clinical data to support the use of 2-DRs in HIV/ TB patients. We chose DTG/3TC for patients with HIV/TB, and HIV-RNA levels < 50 copies/mL in 91.67% of the patients at 48 weeks were observed. Guidelines recommend adjusting the DTG dose to 50 mg twice daily when used with rifampicin,^[20]^ considering patient convenience and reducing economic pressure. One patient used RFB in this study, and the DTG dose was 50 mg once daily. The patient’s TB was successfully treated at 48 weeks, and the patient’s viral suppression effect was improved. Therefore, for patients with TB/HIV, DTG/3TC is a good antiviral treatment option.

Long-term therapy that is safe and efficacious is essential for patients with HIV. In this study, only 1 patient withdrew because of adverse drug events at 4 weeks; therefore, DTG + 3TC has good safety and tolerability. However, our study has some limitations, such as being retrospective, having few people with high VL in the sample selection, and lacking application information for this population.

## 5. Conclusions

DTG + 3TC is a safe option for achieving and maintaining virological suppression in ART-naïve patients with HIV and TB coinfection. Because this regimen has few drug-related adverse events, it ensures increased patient compliance and adherence. However, monitoring patients’ creatinine and lipid levels during long-term ART is necessary.

## Author contributions

**Conceptualization:** Juan Jin.

**Data curation:** Haohua Hou, Huanhuan Ba, Xinyan Jin, Peipei Luo.

**Investigation:** Haohua Hou, Huanhuan Ba, Peipei Luo.

**Resources:** Juan Jin.

**Software:** Haohua Hou, Huanhuan Ba.

**Writing – original draft**: Haohua Hou.

**Writing – review & editing:** Juan Jin.
